# A hard day’s night: time use in shift workers

**DOI:** 10.1186/s12889-019-6766-5

**Published:** 2019-06-03

**Authors:** Tracy L. Kolbe-Alexander, Sjaan Gomersall, Bronwyn Clark, Luciana Torquati, Toby Pavey, Wendy J. Brown

**Affiliations:** 1 School of Health and Wellbeing, University of Southern Queensland, 11 Sallisbury Road, Ipswich, QLD 4305 Australia; 2 School of Human Movement and Nutrition Sciences, The University of Queensland, Brisbane, Australia; 3 Division of Exercise Science and Sports Medicine, University of Cape Town, Cape Town, WC South Africa; 4 School of Health and Rehabilitation Sciences, The University of Queensland, Brisbane, Australia; 5 School of Public Health, The University of Queensland, Brisbane, Australia; 6 School of Exercise and Nutrition Sciences, Queensland University of Technology, Brisbane, Australia

**Keywords:** Shift workers, Time-use, Physical activity

## Abstract

**Background:**

Differences in how shift workers accumulate physical activity (PA) while at work and in leisure time, on days when they are working at night, during the day, or on non-work days, are largely unexplored. The aim of this study was to improve understanding of physical activity patterns in two groups of shift workers, and to measure variations according to their shift schedules.

**Methods:**

This pragmatic pilot study was conducted in two workplaces. Employees in Workplace 1 (*n* = 10) were required to drive for most of their shift. Workplace 2 was a manufacturing company where most of the employees’ (*n* = 30) occupational tasks were completed while standing. Use of time was assessed using the adult version of the Multimedia Activity Recall for Children and Adults (MARCA) administered by telephone interview. Three MARCA interviews were conducted with each participant, in order to capture a typical profile of a day-shift day, a night-shift day and a non-work day, using a two-day recall for each interview. Participants were asked to wear the activPAL3™ activity monitor, for 7 consecutive days. Paired and independent t-tests were used to compute significant differences between day-shift, night-shift and non-work days within and between workplaces.

**Results:**

The total number of days quantified for the MARCA data was 192 days (64 day-shift, 60 night-shift and 68 non-work days). Workplace 2 participants reported more physical activity and less sedentary behaviour on day-shift and night shift days than on non-work days. Time spent in sedentary behaviour was similar on day-shift, night-shift and non-work days in Workplace 1. Workplace 1 participants were more sedentary (*p* = 0.003) and engaged in more light intensity PA (*p* = 0.031) on day-shift and night-shift workdays, than those from Workplace 2. Sleep times were lowest on day-shift days.

**Conclusion:**

As the occupational tasks for participants in Workplace 2 involved physical activities, the findings do not support the conventional view that shift workers are more sedentary than those who only work during the day. Rather occupational tasks appear to be a more important determinant of physical activity patterns both on work and non-work days than varying shift patterns.

## Background

The growing demands of a 24/7 economy have led to growth in the number of people working shifts. Of the 8.6 million employees in Australia, 1.4 million (16%) work shifts. More than half (58%) lack autonomy for choosing their shift times and 30% report working overtime often [1].

Because rates of cardiovascular disease, diabetes and metabolic syndrome are higher in shift workers than in those who only work during the day, shift workers are often referred to as an ‘at risk’ group [2]. This is confirmed by our recent systematic review, which found that the risks of cardiovascular events and coronary heart disease mortality were 17 and 26% higher, respectively, in shift workers than in non-shift workers [3]. Furthermore, there was a dose-response relationship, with a 7% increased risk of cardiovascular disease for every additional 5 years of shift work [3]. Data from a prospective cohort study of 74,862 nurses also show that cardiovascular disease and all-cause mortality are significantly higher in nurses who have worked night shifts for more than 5 years, than in those who never worked night shifts [4].

The role of physical activity in the prevention of non-communicable diseases is well established [5]. Although some shift workers might be aware of the benefits of physical activity, conflicting schedules make it difficult for them to lead physically active lives [6]. The opportunities to participate in leisure-time physical activity are particularly compromised for those who work night or rotating shifts, as the operating hours of many sports and leisure facilities are based around those who work day shifts [6]. Alongside the increased fatigue and disrupted circadian rhythms that occur with shift work, lack of time is a major barrier to the maintenance of regular leisure time physical activity in shift workers [6]. Therefore insufficient levels of physical activity have been identified as one of the potential mechanisms which link shift work with adverse health outcomes [7].

There is however, little evidence that shift workers are less active than those who regularly work only during the day. For example, objective measures of activity in two Danish cohorts show no differences in *leisure-time* physical activity between shift and non-shift workers [7]. In contrast, accelerometer data from the US NHANES study show that, in terms of *overall* physical activity, rotating shift workers do more light intensity physical activity than daytime workers. However, in that study, both evening and night shift workers recorded fewer bouts of work-related moderate-vigorous physical activity than those who worked during the day [8]. Patterns of physical activity in shift workers are therefore complex, and may be further clouded by long periods of sitting time in some occupational groups. Moreover, differences in how shift workers accumulate physical activity while at work and in leisure time, on days when they are working at night, during the day, or on non-work days, are largely unexplored.

Most previous research with shift workers has quantified time spent in physical activities at different intensities, and time spent in sedentary behaviours on work and non-work days [7, 8]. These studies, which rely on accelerometer measures, rarely consider how these activity intensities, or overall patterns of activity, might vary on days when shift workers work at night or during the day. In this paper we therefore used a detailed 24-h recall method to record all activities during day-shift, night-shift and non-work days, and supplemented the data with objective measures of stepping and sitting, to compare overall patterns of time use, and patterns of moving, sitting and sleeping, on day-shift, night-shift and non-work days, in shift workers from two contrasting occupational groups.

The overall aim was to improve understanding of activity patterns on day-shift, night-shift and non-work days in two groups of shift workers. The main objective was to determine the variations in sleep, sedentary behaviour, light and moderate intensity physical activity on day-shift, night-shift and non-work days using validated self-report measures. We hypothesised that shift workers would spend more time sleeping and in moderate to vigorous intensity physical activity on day-shift days than on night-shift days. The second objective was to compare the variations in sleep, sedentary behaviour and physical activity on work and non-work days. Additionally, we explored the differences between activity time-use in workers who experienced contrasting physical demands in their workplaces, to provide direction for future research in shift workers.

## Methods

### Setting and participants

This pragmatic pilot study was conducted in two workplaces in Brisbane, Australia. All employees who worked night or rotating shifts were eligible to participate. Employees in Workplace 1 were required to drive in an airport setting for most of their 12-h shifts and their job demands allowed very little time outside their vehicles, even when not driving. Workplace 2 was a manufacturing company where most of the employees were not office-based and many occupational tasks were completed while standing. The majority of the employees in both workplaces worked 12-h shifts, with day and night shifts separated by non-work days.

### Recruitment

In Workplace 1, the human resources manager identified a single team of employees who worked shifts who would be eligible for participation. Ten of the 30 employees in the team agreed to participate in the research. The Health and Wellness officer in Workplace 2 arranged a series of four seminars to recruit employees from the manufacturing department, as the researchers were not allowed in the factory. Thirty employees attended the seminars and all agreed to participate, however four did not complete all the assessments.

The research was approved by The University of Queensland Human Research Ethics Committee. Participants signed a consent form after researchers delivered a brief presentation on the research. During this visit, each participant arranged three telephone appointments with the researchers for the time-use recall interviews, received their accelerometers, completed a short questionnaire, and had their blood pressure, height and weight measured.

### Measures

#### Multimedia activity recall for children and adults (MARCA)

Use of time was assessed using the adult version of the Multimedia Activity Recall for Children and Adults (MARCA) [9] administered by telephone interview with a trained research assistant. The MARCA is a computerised 24 h recall tool, which asks participants to recall their previous day (24 h) from midnight to midnight in increments as small as 5 min, using meal times as anchor points. Interviewers entered the participants’ activities into the MARCA by choosing from a list of more than 500 activities, organised under a number of drop-down categories including “Self-Care”, “Occupation” and “Sport/Recreation”.

Three MARCA interviews were conducted with each participant, in order to capture a typical profile of a day-shift, night-shift and a non-work day. During each call, the interviewer asked the participant to recall their activities for the two previous days. The mean values for the preceding two days were used in the statistical analysis. The first telephone call was scheduled after two day-shift days, the second call after two night-shift days and the third after two non-work days. The first telephone call took an average of 40 min, with subsequent calls taking approximately 20–30 min each. Previous testing has demonstrated that the MARCA is a reliable and accurate tool, with accuracy superior to most other tools that rely on participant reporting rather than direct measures [10, 11].

#### activPAL3™ measures of sitting, standing and stepping

Participants were asked to wear the activPAL3™ activity monitor, a thigh-worn accelerometer which continuously records posture and movement (time spent sitting/lying, standing or stepping). The activPAL3™ was sealed with a nitrile finger cot and attached to the skin with a transparent hypoallergenic patch, in order to provide a waterproof barrier. The device was placed at the recommended position, one third of the way down the anterior thigh, in the midline. The activPAL devices were initialised and downloaded in PAL version 7.2.32 (PAL Technologies Ltd., Glasgow, Scotland, UK).

Participants were asked to wear the monitor continuously for at least seven days, without removing it, and to only change the hypoallergenic patch when needed. Valid days were defined as those with at least 10 h of estimated ‘awake wear time’ in each wake to sleep period, for each of the three conditions, day-shift, night-shift and non-work day. Participants were asked to provide a log of the hours they worked during the week they wore the activPAL device. From this, day-shift days, night-shift days and non-work days were identified.

#### Questionnaire

The questionnaire asked participants to report their age, gender, marital status, perceived health status, patterns of activity at work, and the impact of shift work on leisure time activities.

#### Clinical measures

##### Blood pressure

Each participant’s systolic and diastolic blood pressures were measured twice using an automated sphygmomanometer [12]. Participants were asked to sit quietly for approximately three minutes before measurements were taken. Both readings were recorded and the mean of the two was used in the statistical analysis.

##### Anthropometry

Standing height (cm) was measured to the nearest 0.1 cm, using a stadiometer (model 217–172-1009, Seca, Hamburg, Germany) [12]. Body weight was measured using a portable calibrated scale (Model MS 3200, Charder Hamburg, Germany) and recorded to the nearest 0.1 kg [12]. Participants were asked to remove shoes, jackets and to empty pockets for these measurements. Body Mass Index (BMI) was calculated as body mass in kg divided by height in m squared (kg/m^2^).

#### Data management and statistical analysis

##### MARCA

Each reported activity was classified into one of ten ‘super domains’ of time-use: Sleep; Quiet Time; Screen Time; Self-Care; Chores; Work/Study; Social; Cultural; Transport; or Physical Activity [13]. These were linked to a compendium which included details of each activity, including body posture (lying, sitting, standing or locomotion), and estimated energy expenditure in metabolic equivalents (METs), based largely on the Ainsworth Compendium of Physical Activities [14]. Mean (SD) time spent in each super domain during day-shift, night-shift and non-work days was calculated for both occupational groups. Mean (SD) durations (min/day) of sleep, sedentary behaviour, and light, moderate and vigorous intensity activities were also calculated for each day type (day-shift, night-shift and non-work day). Sleep time was determined by summing the duration of all sleep episodes (including napping), and time in sedentary behaviours was determined by summing the duration of activities at < 1.5 METs in a sitting or lying posture whilst awake [15]. Time spent in light, moderate and vigorous physical activities was determined by summing the duration of activities at1.5 - <3METS, ≥3 - < 6 METs, and ≥ 6 METs respectively. All statistical analysis used an average of the data reported in two recalls. The proportions of daily time spent in each activity domain were calculated using 24 h (1440 min) as the denominator.

##### activPAL

Days were determined as time from waking up to going to sleep with periods of sleep identified in the data using a validated algorithm [16]. Durations of time spent sitting, sitting in bouts of ≥30 min (prolonged sitting), standing, and stepping were calculated using the activPAL events files. These were recorded as total time per day, and as percentage of awake wear time per day, using ‘awake time’ for that day as the denominator.

Means (Standard Deviation) were calculated for all continuous variables including age, BMI and durations of all activities recorded by the MARCA and activPAL. All data were checked for normality using the Shapiro-Wilk test. Non-normally distributed data were log transformed and parametric statistics were performed. Independent and paired t-tests were used to compute significant differences between workplaces and between day-shift, night-shift and non-work days. Frequencies and proportions were computed for categorical variables, and Chi squared analyses were used to determine significant differences between groups. All statistical analyses were conducted in SPSS version 23. *P*-values were based on two-sided tests and were considered statistically significant at *p* < 0.008 for Workplace 1 and *p* < 0.008 for Workplace 2.

## Results

### Participant characteristics

Characteristics of the participants, in the two occupational groups (Workplace 1; *n* = 10, and Workplace 2, *n* = 30), are shown in Table 1. Most were middle aged, married men with good to excellent self-rated health. Mean BMI was in the overweight range in both the airport (Workplace 1) and manufacturing workers (Workplace 2), but elevated blood pressure was more common in the latter group. The airport workers reported their occupational movement pattern as ‘mostly sitting’, while more than half the manufacturing workers reported mostly moderate intensity activity, with some periods of vigorous activity at work.

**Table 1:** Demographic and health characteristics of the participants

	Workplace 1 (*n* = 10)	Workplace 2 (*n* = 26)
	*mean ± SD*	*mean ± SD*
Age (years)	41.30 ± 6.27	46.17 ± 8.67
Body Mass Index (kg/m^2^)	26.77 ± 3.12	29.55 ± 4.41
Systolic Blood Pressure (mm Hg)	123.29 ± 9.86^#^	140.81 ± 13.57^#^
Diastolic Blood Pressure (mmHg)	74.29 ± 2.14^#^	90.18 ± 14.00^#^
	***%***	***%***
Sex		
Male	70	100
Marital Status		
Married/living with partner	90	88
Self-reported Health Status		
Excellent	0	17
Good	70	50
Average	30	29
Poor	0	4
Movement Pattern at work		
Mostly sitting	100	18^#^
Mostly standing still	0	0
Mostly walking for short periods	0	27^#^
Mostly mod PA / vig PA for short periods	0	55^#^

^#^Difference between workplace 1 and workplace 2, *p* < 0.05

#### Activity patterns from the MARCA data

**Fig. 1:**
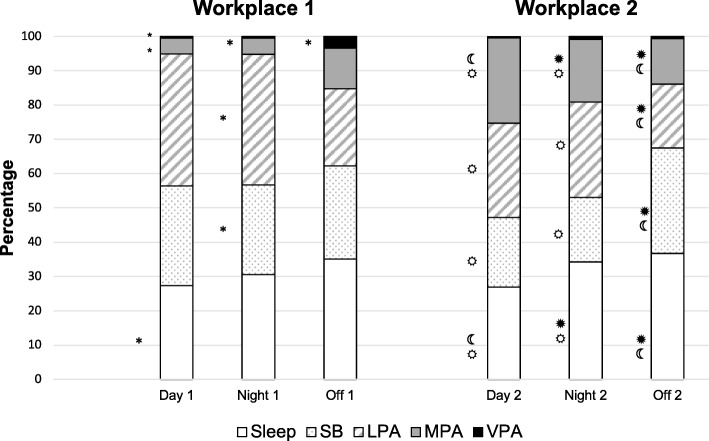
Percentage of time in sleep, sedentary behaviour, light, moderate and vigorous intensity physical activity (MARCA). SB: sedentary behaviour; LPA: light intensity physical activity; MPA: Moderate intensity physical activity; VPA: vigorous intensity physical activity. * = Significant difference between Workplace 1 and Workplace 2, *p* < 0.05, using independent t-test. ^☼^ Different from non-work days, *p* < 0.008; ^☀^Different from day shift days, *p* < 0.05; ^☾^ Different from night shift days; using paired t-test; Workplace 1. ^☼^ Different from non-work days, *p* < 0.05; ^☀^Different from day shift days, *p* < 0.05; ^☾^ Different from night shift days; using paired t-test; Workplace 2

The total number of days quantified for the MARCA data was 192 days, comprising 64 day-shift, 60 night-shift and 68 non-work days. Of the 10 participants in Workplace 1, eight had complete MARCA data for day-shift, and nine for night-shift and non-work days. Of the 30 Workplace 2 participants, 24 reported on their day-shift time use, 21 reported on night-shift and 25 on non-work days. Because the activity and occupational tasks for the two workplaces were significantly different, and the small sample size in Workplace 1, the data are presented separately for each workplace. The proportions of time spent in sleep, sedentary behaviour, light, moderate and vigorous intensity physical activity, derived from the MARCA data (self-reported) and MET values for each super domain, for the two workplaces are shown in Fig. 1.

##### Comparisons of day-shift, night-shift and non-work days in each workplace

Among Workplace 2 participants, there were significant differences in sleep, sedentary behaviour and physical activity on day-shift, night-shift and non-work days. The proportion of time spent in moderate intensity physical activity was significantly higher for these workers on day-shift days (25%) than on night-shift (18%; *p* = 0.042) and on non-work days (19%;*p* < 0.001). These participants also reported more sedentary behaviour on their non-work days (37) than on either day-shift (20%) or night-shift days (19%; *p* < 0.001), however the proportion of time spent sedentary was similar on day and night shifts. On the other hand, the participants spent significantly less time in light intensity physical activity on their non-work days than on work days, 31% versus 28%, *p* < 0.009. Sleep time was highest on non-work days and lowest on day-shift days (*p* < 0.03). Overall, therefore, these workers reported more physical activity and less sedentary behaviour on their work days than their non-workdays. In workplace 2, the high level of sedentary behaviour on non-work days was offset by less time in light and moderate intensity physical activities.

Despite the participants in Workplace 1 reporting more moderate and vigorous intensity physical activity on non-work days than when working day or night-shifts, these differences were not significant (significance for this group was *p* < 0.008). The proportion of time spent sleeping, sedentary and in light-moderate-and vigorous intensity was similar when working day-shift and night-shifts. There was no difference in sedentary behaviour time on work and non-work days for these participants.

**Fig. 2:**
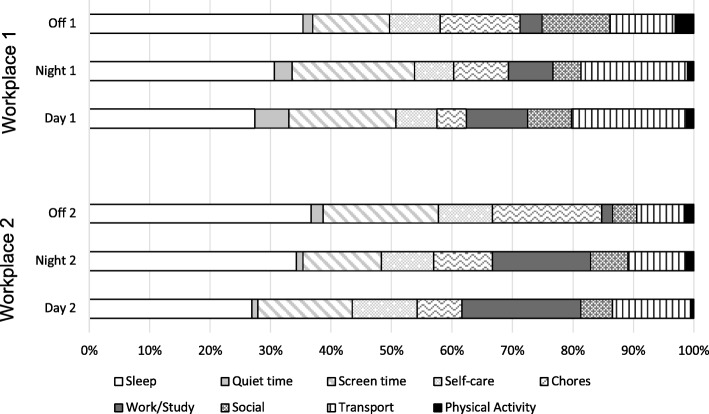
Proportions of time spent in each MARCA super domain for Workplace 1 and Workplace 2. Off1 = Workplace 1 non-work day; Night 1 = Workplace 1 night shift; Day 1 = Workplace 1 day shift; Off2 = Workplace 2non-work day; Night 2 = Workplace 2 night shift; Day 2 = Workplace 2 day shift

Mean (SD) time spent in each of the ten MARCA super domains of time use are shown in Table 2 for participants in each workplace. These data are also shown as proportions of a 24 h day in Fig. 2.

**Table 2:** Time (min/day) spent in all super domains during day-shift, night-shift and non-work days [MARCA data, mean (standard deviation)]

Super Domain	Workplace 1			Workplace 2		
	Day shift (*n* = 8)	Night Shift (*n* = 9)	Non-work day (*n* = 9)	Day shift (*n* = 25)	Night Shift (*n* = 22)	Non-work day (*n* = 26)
Sleep	393.5 (56.32)^☼^	439.9 (72.27)^☼^	505.4 (53.00)^☾☀^	387.3 (61.40)^☾☼^	493.3 (92.86)^☀☼^	528.6 (91.33) ^☾☀^
Quiet time	80.0 (133.15) ^#^	42.5 (40.65) ^#^	23.2 (27.97)	14.0 (32.71)^#^	15.8 (23.17)	28.6 (46.01)
Screen time	255.2 (205.31)	292.0 (111.54)^#^	181.6 (101.20)	225.2 (164.08)	186.7 (121.62)^#☼^	274.6 (147.71)^☾^
Self-care	97.1 (31.96) ^#^	92.8 (21.62)^#^	119.9 (31.20)	154.0 (40.06)^#☾^	123.8 (28.88)^#☀^	128.6 (40.88)
Chores	69.9 (46.51) ^☼^	130.4 (86.35)	189.7 (109.90)^☀^	107.3 (110.24)^☼^	140.8 (125.77)^☼^	260.0 (121.40)^☾☀^
Work / study	146.1 (107.44)	106.2 (59.01)	52.0 (73.67)	283.5 (184.42)	234.0 (151.96)^#^	25.8 (46.63)
Social	104.2 (78.40)	67.0 (68.8)	160.2 (105.54)^#^	75.6 (64.23)	88.3 (86.75)	58.1 (82.60)^#^
Cultural	0 (0)	0 (0)	0.3 (0.83)	0 (0)	0 (0)	0 (0)
Physical Activity	17.4 (17.91)	14.7 (26.57)	44.1 (65.40)	8.0 (14.82)	18.6 (33.08)	22.9 (36.51)

^#^Difference between workplace 1 and workplace 2, *p* < 0.05 using independent t-tests

^☼^ Different from non-work days, *p* < 0.008; ^☀^Different from day shift days, *p* < 0.05; ^☾^ Different from night shift days; using paired t-test; Workplace 1

^☼^ Different from non-work days, *p* < 0.05; ^☀^Different from day shift days, *p* < 0.05; ^☾^ Different from night shift days; using paired t-test; Workplace 2

In Workplace 2, screen time was lowest on night-shift days (3.75 h) and highest on non-work days (4.6 h, *p* = 0.014). These participants reported spending more time doing chores on their non-work days than when working day-shifts (*p* = 0.005) or night-shifts (*p* < 0.001). They spent approximately 30 min more each day doing chores, and 30 mins less each day in self-care activities on night-shift days than on day-shift days. These participants also spent more time in self-care activities on day-shift days than night-shift days. Compared with both night-shift and non-work days, self-reported physical activity was lowest when on day-shifts, however these differences were not significant.

In Workplace 1, there were no significant differences in any of the MARCA super-domains for day-shift, night-shift and non-work days (Table 2). Similar to Workplace 2, the participants in Workplace 1 reported more time doing chores on their non-work days than on day-shift (*p* = 0.043) and night-shift (0.017) days. These participants reported most physical activity on their non-work days (44 min per day) than their day-shift (17 min per day) and night-shift days (15 min per day).

##### Comparison of two workplaces

The participants in Workplace 1 were significantly more sedentary (*p* = 0.003) and engaged in more light intensity physical activity (*p* = 0.031) on day-shift and night-shift days than those from Workplace 2. Conversely, those in Workplace 2 engaged in significantly more moderate intensity physical activity, on both day-shift (*p* = 0.01) and night-shift (*p* = 0.04) days than on non-work days. Workplace 1 participants reported significantly more vigorous intensity physical activity on non-work days than those from Workplace 2. However, when moderate and vigorous intensity physical activity were considered together, there were no differences between the two workplaces on non-work days.

Participants in Workplace 1 reported significantly more time in ‘quiet time’ activities on day-shift workdays (*p* < 0.001), and in transport-related activities (*p* = 0.012) and screen time (*p* = 0.034) on night-shift days, than those in Workplace 2. Conversely, participants in Workplace 2 reported significantly more time in ‘self-care activities’ on both day-shift and night-shift days (*p* < 0.001), and significantly more ‘work/study’ time (*p* = 0.03) on night-shift days, than those from Workplace 1. Time spent in cultural-related activities was minimal in both workplaces.

### activPAL measures of physical activity and sedentary behaviour

Mean duration (and percentages of awake time), spent in sitting, standing and stepping activities, derived from the activPAL records, are shown in Table 3.

**Table 3:** Mean (SD) time and percentage of awake time spent in various activities on day-shift, night-shift and non-work days, as determined by activPAL3

	Workplace 1			Workplace 2		
	Day shift (*n* = 7)	Night Shift (*n* = 5)	Non-work day (*n* = 6)	Day shift (*n* = 19)	Night Shift (*n* = 16)	Non-work day (*n* = 16)
Valid Days: per person Median (IQR)	2.6 (1.5; 2.5)	2.2 (2.0;2.5)	3.0 (3.0; 3.5)	2.0 (1.0;4.0)	3.0 (3.0;3.0)	2.7 (2.0;4.0)
Awake (hours per day)	16.5 ± 1.96^#^	17.6 ± 3.13^#^	15.3 ± 1.31	13.4 ± 2.61^#☾^	22.2 ± 3.66^#☼☀^	15.2 ± 1.89^☾^
Sitting						
Hrs/day	11.1 ± 2.80^#^	12.0 ± 3.73	9.2 ± 2.05	6.5 ± 2.43^#☾☼^	11.4 ± 2.72^☼☀^	8.2 ± 1.55^☾☀^
% awake	66.6 ± 12.79^#^	66.4 ± 10.39^#^	59.9 ± 10.29	47.24 ± 12.72^#^	51.4 ± 8.31^#^	54.5 ± 10.42
Sitting (in bouts ≥ 30 min)						
Hrs/day	4.0 ± 2.19^#^	5.4 ± 2.48	4.4 ± 2.20	2.4 ± 1.49^#☾☼^	4.7 ± 2.72^☀^	3.8 ± 1.96^☀^
% awake	24.08 ± 12.32	29.75 ± 10.21	28.0 ± 12.48	17.3 ± 9.74	20.6 ± 9.64	24.8 ± 13.21
Standing						
Hrs/day	3.8 ± 1.41	4.0 ± 0.86^#^	4.0 ± 1.03	4.8 ± 1.32^☾^	7.2 ± 2.06^#☼☀^	4.6 ± 1.40^☾^
% awake	23.21 ± 10.14^#^	24.00 ± 9.65^#^	26.3 ± 6.62	36.4 ± 10.03^#^	32.5 ± 6.33^#^	30.32 ± 8.27
Stepping						
Hrs/day	1.7 ± 0.60^☼^	1.7 ± 0.40^#^	2.1 ± 0.76^☀^	2.2 ± 0.79^☾^	3.6 ± 1.15^#☼☀^	2.4 ± 1.08^☾^
% awake	10.2 ± 3.67^#☼^	9.6 ± 2.23^#^	13.8 ± 5.43^☀^	16.4 ± 5.16^#^	16.1 ± 4.23 ^#^	15.2 ± 5.33
Steps (n/day)	3872 (511.87) ^☼^	3938 (421.58)^#^	4739 (796.34) ^☀^	5229 (392.06)^☾^	8275 (628.86)^#☀☼^	5207 (578.33)^☾^

IQR: 25th and 75th percentiles

^#^Difference between workplace 1 and workplace 2, *p* < 0.05 using independent t-test; ^☼^ Different from non-work day, *p* < 0.05; ^☀^Different from day shift days, *p* < 0.05; ^☾^ Different from night shift days *p* < 0.05 using paired t-test statistics

#### Comparisons of day-shift, night-shift and non-work days in each workplace

Objectively measured time spent in activity and sedentary behaviour was largely similar on day-shift, night-shift and non-work days for participants in Workplace 1 (Table 3). The only significant difference was for time spent stepping, which was higher on day-shift than on non-work days (*p* = 0.002).

Workplace 2 participants had significantly more awake time on night-shift than on day-shift and non-work days (*p* < 0.001). Significantly more time was therefore spent sitting (*p* < 0.001), sitting in bouts of 30 min or more (*p* = 0.02), standing (*p* < 0.001) and stepping (*p* = 0.001) on night-shift days than on other days. The average number of steps accumulated per day was also significantly higher on night-shift (8275 steps) than on day shift (5229 steps) or non-work days (5207 steps) in Workplace 2 (*p* = 0.03). Time spent sitting, sitting in bouts longer than 30mintues and standing were significantly higher on night-shift days (4.7 h) than on day-shift days (2.4 h per day; *p* = 0.001). These differences were no longer significant when the proportion of time sitting, standing or stepping as a percentage of awake time were compared.

#### Comparison of the two workplaces

Wake time was significantly higher on day-shift days and significantly lower on night-shift days in participants from Workplace 1 than in those from Workplace 2 (*p* = 0.02). Times spent sitting (hours per day) (*p* < 0.001) and sitting in bouts lasting more than 30 min (*p* = 0.038), were significantly higher on day-shift days in Workplace 1 (than in Workplace 2). The proportion of time spent sitting was also significantly higher on both day- and night-shift days for Workplace 1 participants (than Workplace 2; *p* = 0.03). Thus the activPAL data confirm the sedentary nature of occupational tasks in Workplace 1.

Participants in Workplace 2 spent significantly more time standing (in hours per day) on night-shift days (*p* = 0.01), and the proportions of awake time spent standing were significantly higher on both day-shift and night-shift days, than in Workplace 1 participants. The proportion of awake time spent stepping was also significantly higher on day-shift and night-shift days for participants in Workplace 2, who also recorded significantly more steps on night-shift days, than the Workplace 1 participants.

## Discussion

The aim of this pragmatic pilot study was to compare time use patterns on day-shift, night-shift and non-work days in shift workers from two workplaces. We hypothesised that shift workers would spend more time sleeping and being physically active on day-shift days than on night shifts or on non-work days. This hypothesis was partially supported by the data from Workplace 2 which showed significantly more moderate intensity physical activity on day-shift days than on night shift and non-work days. However, sleep duration, based on self-report data, was highest on night-shift days (compared with day-shift and non-work days) in Workplace 2, which did not support our hypothesis. Furthermore, our participants were more sedentary on their non-work days than on day-shift or night-shift days.

This study compared various self-reported activities described in the MARCA for day-shift, night-shift and non-work days in the two groups of shift workers. There are no other similar studies in shift workers, however comparisons with other studies of adults who worked during the day are possible. The shift workers in our study reported more leisure time physical activity, than day workers in another Australian study [17]. Those in Workplace 2 had less quiet time, but more self-care and chores on their non-work days than those who only worked during the day [17]. The differences in time for each of the super-domains were more marked between workplaces, than for the shift schedule within each workplace. Screen time was higher in Workplace 1, however the average time exceeded 3 h per day for all the participants. Displacing this screen time with physical activity could be a potential strategy for future interventions.

Employees in Workplace 1 spent most of their shifts driving vehicles, while those in Workplace 2 were based in a manufacturing company and some of their tasks included standing and moving. Consequently, the participants in Workplace 1 perceived their occupations to be predominantly sedentary, whereas those in Workplace 2 reported that their work tasks required more standing and moderate intensity activity. Therefore, the Workplace 2 participants moved significantly more and sat significantly less on workdays, irrespective of shift, than those from Workplace 1. Our findings are in line with previous research which shows that work-related tasks and classification are determinants of time spent physically active and sedentary. For example, previous studies have shown that those employed in physically demanding occupations spent less time sedentary, both in total and at work, than those in white collar occupations [18]. This is similar to Peplonska et al’s findings who reported that occupational activity contributed more than leisure time physical activity to total daily energy expenditure in nurses and midwives [19].

When analysing the MARCA data according to METs, our participants compensated for their occupational physical activity on non-work days, but in different ways. The airport employees (Workplace 1) reported more physical activity on their non-work days. In contrast, the manufacturing employees (Workplace 2) whose work included moderate intensity physical activity, spent more time sedentary and less time physically active on their non-work days than on workdays. Our findings are different from previous research which has shown that employees with more sedentary occupations compensate this behaviour by engaging in more light or moderate intensity physical activities on non-work days [18, 20]. A cross-sectional study investigating work and leisure time sitting in Australian employees, including shift workers, found that those with sedentary occupations did not sit significantly less during leisure time and vice versa [18]. The results of the cross-sectional study also showed that working more than eight hours per day was associated with less leisure-time sedentary behaviour. Most of our participants worked for more than eight hours per shift, which might, in part, explain the compensatory behaviour we observed in our study.

The occupational tasks for participants in Workplace 2 were physically active, which does not support the conventional view that shift workers are more sedentary than those who only work during the day. Hulsegge and co-workers compared occupational and leisure time physical activity in shift workers and non-shift workers [7]. These Dutch shift workers were more sedentary at work than the non-shift workers, but had similar amounts of leisure time physical activity. Another study which included participants from the National Health and Nutrition Examination Survey (NHANES) reported that those who worked evening and night shifts were less physically active than those who only worked during the day [8]. Unlike our study, the NHANES participants’ professions were unknown. It is therefore plausible that most of the NHANES participants were employed in occupations that were similar to our Workplace 1 participants, resulting in lower levels of occupational and total moderate to vigorous intensity activity.

Workplace 2 participants reported more leisure-time physical activity on non-work days (MARCA super domain), however this was only 22 min per day. The airport workers (Workplace 1) were also more active on non-work days, reporting almost twice as much leisure-time physical activity than those in Workplace 2. However, the total self-reported moderate to vigorous intensity activity on non-work days was similar for participants in the two workplaces. This finding was supported by our activPAL data which showed that there were no differences in total steps per day between the workplaces on a non-work day. These findings are in line with other research which shows that while variations in occupational related activity might be large, leisure-time physical activity is similar for those who work full time [21]. Our findings underscore the contribution of work-related physical activity to total energy expenditure and raises the issue of why this work-related activity is not usually ‘counted’ in population surveillance of activity levels. Most population surveys ask about transport and leisure time physical activity, which in our participants, accounted for a very small proportion of daily energy expenditure. It is not surprising therefore that our participants would be categorised as ‘insufficiently’ physically active.

Self-reported sedentary behaviour was highest on non-work days and lowest on night-shift than on day-shift in Workplace 2. In Workplace 1, participants’ sedentary time was similar on work and non-work days. However, the accelerometer data for the Workplace 1 participants showed that they spent less time in sedentary behaviour on non-work days than on workdays. This finding is in contrast with that of Wong and colleagues’ who used Actigraph GT3x accelerometers to measure activity patterns on work and non-work days. They showed that bus drivers spent a significantly higher proportion of time sedentary on a non-work day than workdays [20]. One of the reasons for the difference between our finding and Wong et al’s, might be that the bus drivers occupational tasks included incidental activity when inspecting their vehicle or loading/unlading the bus [20]. Indeed, the proportion of time spent sedentary was much higher in our Workplace 1 participants (67%) than in the bus drivers (52%) whereas the proportion of time spent sedentary on non-work days was similar (60 and 64%) in the two studies. Regardless of their work tasks or shifts, it would appear that there is a case for encouraging employees to decrease their sedentary behaviour on non-work days. Interventions targeting employees who stand and move at work must include strategies to encourage them to decrease sedentary behaviour on non-work days.

Sleeping less than seven hours per night is associated with adverse health outcomes such as increased risk for cardio metabolic disease [22]. The participants in our study had sufficient sleep on night-shift and non-work days, but not on day-shift days. Increased sleep duration on non-work days is expected, and similar to findings from other studies that have investigated sleep duration in shift workers [23, 24]. One the other hand, sleeping more on night-shift days was unexpected, as we hypothesised that shift workers would sleep less when working night shift. Our hypothesis was based on evidence from a systematic review investigating the effects of shift work on sleep, which showed that the average sleep duration is 1–4 h shorter when working night shifts [25]. The average age of our participants was 44 years which suggests that they might have been working shifts for multiple years and have adapted to the demands of shift work resulting in improved sleep duration, even when working night shifts. The participants in our study reported 7.3–8.2 h of sleep per night, when working night-shifts. This is consistent with sleep guidelines, however these data are based on 2–4 nights of sleep and might not reflect habitual patterns.

The average sleep duration for our participants was more than that observed in other studies. For example, nurses who worked 12-h night and day shifts, which is similar to most of our participants’ shift pattern, reported less than 6 h of sleep per night when working shifts [26]. These nurses also reported little difference in sleep duration between night and day shifts. The nurses were all female and only 22% worked rotating shifts, whereas most of the participants in our study were males, and all had rotating shifts. Female shift workers are more likely to have additional domestic and care-giving responsibilities, restricting their opportunity to sleep, especially when working night shifts [27] . Although we don’t know the exact differences in shift rosters between our participants and the nurses, the variation in shift patterns, and gender, could influence sleep duration on work and non-work days. In contrast with the MARCA data which showed higher sleep times on night-shift days, the activPaL data showed that awake time was higher on night-shift than on day-shift or non-work days, especially in Workplace 2. This apparent contradiction may reflect inaccuracies with self-report data, but may also reflect the fact that ‘days’ were derived differently when using the two measures.. For the MARCA, time spent in each of the activities was quantified from midnight to midnight, whereas the activPAL data were based only on awake time.

### Strengths and limitations

This is one of the first studies to compare detailed time use patterns, across different scenarios in rotating shift workers using both self-report and objective measures. Because recruitment was determined by the workplaces, and we were unable to approach employees at their workstations and invite them to participate, participant numbers were relatively low, especially for Workplace 1. This limited our ability to compare the two workplaces, and our finding should be considered in light of this being a pragmatic, pilot study. The small sample size also limits the generalisability of our findings to shift workers in other industries.

The main data presented were obtained from self-reported time use (MARCA) with a potential for recall bias and error. However MARCA data have previously been shown to correlate strongly with objective measures of physical activity and sitting time [9]. As indicated above, there were challenges in comparing the MARCA (subjective) and activPAL (objective) because of the data collection methods used. For example, participants recalled activities in 5-min epochs for the MARCA for 24 h, whereas the data from the activPAL were averaged over one minute epochs for time spent awake.

Notwithstanding the MARCA provides in-depth and detailed information on day-shift, night-shift and non-work days for domains which include social activities, chores and cultural activities which are not routinely quantified in shift work research.

## Conclusion

Our findings show demonstrate patterns of moving, sitting and sleeping on day-shift, night-shift and non-workdays in two small groups of blue collar workers. Differences in moving and sitting patterns were more marked when the two workplaces were compared with each other, than when day and shift types were compared within each group. In Workplace 1, the employees were more sedentary and did less light intensity activity on workdays, but reported more vigorous intensity physical activity on non-work days. The Workplace 2 participants were more physically active on their workdays and reported less moderate and vigorous intensity physical activity on their non-work days, suggesting that there was some compensation for occupational-related activity.

Therefore patterns of moving and sitting might be more affected by the type of occupation and associated tasks, than by the shift being worked, but follow up-research is needed to confirm the results of this small study. Future interventions should aim to increase workplace- activity, especially among those with more sedentary occupations. This might be more important for shift workers, like those in Workplace 1, who have predominantly sedentary occupations and limited opportunity for occupational and leisure time physical activity. Conversely, employees who perceive their occupational tasks to be physically active, might opt to spend more time sedentary during their leisure time and should be encouraged to increase their leisure time physical activity.

## Abbreviations

BMI: Body Mass Index
MARCA: Multimedia Activity Recall for Children and Adults
PA: Physical activity
SD: Standard Deviation
US NHANES: United States National Health and Nutrition Examination Survey
